# Primary Health Care: Our Experience From an Urban Primary Health Care Center in Greece

**DOI:** 10.7759/cureus.35241

**Published:** 2023-02-20

**Authors:** Spyridon P Galanakos, George D Bablekos, Chara Tzavara, Nikolaos D Karakousis, Eleftherios Sigalos

**Affiliations:** 1 Primary Health Care, Orthopedics and Trauma, Alexandra Health Center, Athens, GRC; 2 Biomedical Sciences, Occupational Therapy and Nursing, University of West Attica, Athens, GRC; 3 Biostatistics, National and Kapodistrian University of Athens, Athens, GRC; 4 Primary Health Care and Internal Medicine, Amarousion Health Center, Athens, GRC; 5 Primary Health Care and Radiology, Alexandra Health Center, Athens, GRC

**Keywords:** emergency medical service, public health, health services, epidemiology, primary health care

## Abstract

This observational study reported patient data derived from the emergency files of a primary health care (PHC) center in Greece, with the aim of providing potential solutions for a well-organized, well-structured, and effective social healthcare system.

This series was conducted at a single urban PHC center in Greece between August 2017 and March 2020. A total of 83,592 patient visits were registered. The mean patient age was 42.5 years (range: three months to 93 years). Demographics, presenting complaints, and the need for patients who visited the healthcare center to be referred to tertiary hospitals were examined. Further perspectives and future strategies to strengthen the national PHC system were addressed.

The most common reasons for visits were pathological (33.6%), followed by general surgery (21.2%) and orthopedics (18.1%). Pediatric conditions accounted for 12% of visits, cardiological conditions accounted for 8.6%, and dental problems accounted for 6.8%. The majority of the patients (n = 81,317, 97.3%) were managed within the health center, and only 2.7% of cases (n = 2275) needed to be referred to a secondary or tertiary healthcare structure. Reasons for patient referral included the severity or complexity of the patient's situation, lack of a specific medical specialty, and the unavailability of overnight laboratory tests.

The PHC center remains the cornerstone of a high-quality healthcare system. A well-structured PHC unit can improve health outcomes and decongest secondary and tertiary health care.

## Introduction

Primary health care (PHC) is defined as a system that provides a bundle of basic and complete healthcare services at the individual and family levels and constitutes the first contact point of citizens with the health system [1]. A general impression is that the PHC system in Greece has failed to reach its targets, especially continuity of care, integrated and coordinated care, and patient- and family-focused care [2]. Among these difficulties in Greece, many attempts have been made to overcome all the adversities to improve the efficiency of PHC; yet, it is still an issue of debate and remains a difficult issue among policy-makers, providers, and primary care practitioners [3].

In our country, by a specific law in 2017, Alexandra Medical Center was given the responsibility of serving as a PHC center for patients who need emergency management in an essential medical manner. The number of patients served by this center in the middle of Attica, Greece, is approximately one million. This public service is accessible 24 hours a day, seven days a week (24/7), providing internists or general practitioners, pediatricians, cardiologists, and nursing personnel. General surgeons, orthopedic surgeons, biopathologists, dentists, and radiologists, who also provide services, are present in the morning (08:00 AM-15:00 PM) and afternoon (15:00 PM-22:00 PM) and are on call during nights (22:00 PM-07:59 AM).

To the best of our knowledge, a study focused on patient visits and management by the first urban PHC center in Greece with an emergency department working 24 hours a day has not been discussed in the related literature. Therefore, we performed this study to describe the characteristics of people who visited the Alexandra Avenue healthcare center, the presenting complaints, and the need for patient transfer to tertiary hospitals. In addition, further perspectives and future strategies were mentioned for strengthening the national PHC system.

## Materials and methods

Setting and study design

This observational study was conducted at a single urban PHC unit. The emergency room of our unit is a level III trauma center, which has demonstrated the ability to provide prompt assessment, resuscitation, surgery, intensive care, and stabilization of injured patients and emergency operations.

The population surveyed included patients who visited the healthcare unit to receive health care regarding pathology, cardiology, pediatrics, or trauma-related issues.

Data collection and processing

Demographic and clinical data for each patient were registered in an electronic medical record, and the examined parameters of the study were retrieved from the visit record.

Data were retrospectively collected from the prospectively functioning electronic system of the PHC center, from August 2017 to March 2020. We extracted information pertaining to a variety of patient attributes: age, race or ethnicity, primary language spoken, mode of arrival, the reason for visit, diagnosis, medical services provided, and the need for referral to a tertiary (third-level) hospital. Additionally, we communicated with the hospital that received the referral to extract the validity of our suspected diagnosis, as stated in the referral letter, for the final diagnosis.

Statistical analysis

The following data points were recorded: emergency department (ED) visits, time of attendance (morning, afternoon, night), and specialty referral/time period (month) of visit. Descriptive statistics were calculated for all collected variables. Pearson correlation analysis was performed considering all variables as continuous measurements with a Gaussian distribution.

R-values (Pearson correlation analysis) and their 95% confidence intervals were computed for each data point per specialty against the total number of referrals across all months, for each month per specialty against the total number of visits during the respective month, and for each data point per time period (afternoon: 15:00 AM-22:00 PM and night: 22:00 PM-8:00 AM) of patient arrival against the peak time visits (morning: 8:00 AM-15:00 PM) across all months.

Paired student’s t-tests were used to compare the number of patients who were attended during the morning shift with the number of patients who were attended during the afternoon and night shifts. The chi-square test for homogeneity was used to examine the changes in the number of patients who were attended in each specialty within the follow-up period.

All reported p-values were two-tailed. Statistical significance was set at p < 0.05, and analyses were conducted using SPSS statistical software (IBM SPSS Statistics for Windows, Version 22.0; IBM Corp., Armonk, NY).

## Results

In this study, we sought to analyze the potential benefits of a strengthened primary care sector in the Greek healthcare system by analyzing data from patients who visited the selected reference primary care center located in the center of Athens within a time span of 33 months. Our data collection spanned between August 2017 and March 2020. A total of 83,592 visits occurred during the study period, with a median of 2,617 cases per month. The mean age was 42.5 years, with a minimum recorded value of three months and a maximum of 93 years. Patients were mostly Greek nationals (81%), followed by 10% from the Balkans and 9% from other countries (Egypt, Iran, Iraq, Saudi Arabia, Syria, Afghanistan, Pakistan, and India). The most common languages were Greek, Arabic, and English.

The most common presenting complaints at the ED visits were pathological conditions (34.3%), followed by general surgical (21.3%) and orthopedic conditions (16.7%). Pediatric conditions accounted for 12.4% of visits, cardiological conditions accounted for 8.8%, and dental problems accounted for 6.4% (Table 1).

**Table 1:** Causes of emergency department (ED) visits per specialty during the study period

Pathology (n = 28,666)	Cardiology (n = 7399)	Pediatric (n = 10,384)	General surgery (n = 17,832)	Orthopedic (n = 13,966)	Dentistry (n = 5345)
Respiratory diseases (7815: 27.2%)	Hypertension (2615: 35.3%)	Fever (3230: 31.1%)	Trauma (8554: 48%)	Low back/cervical spine pain (5062: 36.2%)	Toothache (2183: 40.8%)
Digestive diseases (6837: 23.8%)	Chest pain (1944: 26.3%)	Respiratory diseases (1982: 19.1%)	Abdominal pain (5165: 29%)	Tendinitis (3471: 24.8%)	Aphthous ulcer (1286: 24%)
Urogenital diseases (5749: 20%)	Cardiac arrhythmias (1857: 25.1%)	Abdominal pain (1503: 14.5%)	Burns (1426: 8%)	Sprains (3421: 24.5%)	Bleeding gums (697: 13%)
Allergies (5026: 17.6%)	Other (983: 13.3%)	Diarrhea (1255: 12.1%)	Physical assaults (1193: 6.7%)	Arthritis (1483: 10.6%)	Injury to mouth or jaw (404: 7.6%)
Other (3239: 11.4%)		Vomiting (1083: 10.4%)	Peripheral vascular disorders (477: 2.6%)	Fractures (459: 3.3%)	Abscess (364: 6.8%)
		Allergies (1048: 10.1%)	Other (1017: 5.7%)	Dislocations (15: 0.2%)	Tooth fracture (162: 3.1%)
		Other (283: 2.7%)		Other (55: 0.4%)	Other (249: 4.7%)

To understand whether there was a continuous trend among patient needs for a particular specialty, we sought to break down the influx of patients per specialty/month. A similar trend was observed across all months without any significant deviations per specialty (p > 0.999). The specialty with the highest demand was, as expected, pathology. All specialties displayed a positive correlation with that trend, e.g., as the number of visits increased, there was a proportional distribution among specialties, considering the analogous demand (Table 2).

**Table 2:** R-values (correlation analysis) were computed for each datapoint per specialty against the total number of referrals across all months and per time period (afternoon: 15:00 AM-22:00 PM and night: 22:00 PM-8:00 AM) of patient arrival against the peak time visits (morning: 8:00 AM-15:00 PM) across all months. Two-tailed p-values were calculated for each Pearson correlation coefficient, with the confidence interval set at 95%.

	Total vs. Pathology	Total vs. Cardiology	Total vs. Pediatrics	Total vs. Surgery	Total vs. Orthopedics	Total vs. Dentistry	Morning 8:00-15:00 vs. Afternoon 15:00-22:00	Morning 8:00-15:00 vs. Night 22:00-8:00
Pearson r								
r	0.929	0.801	0.898	0.663	0.720	0.486	0.932	0.889
95% confidence interval	0.791; 1.000	0.578; 1.000	0.734; 1.000	0.384; 0.942	0.461; 0.979	0.160; 0.812	0.797; 1.000	0.718; 1.000
R squared	0.863	0.642	0.806	0.440	0.518	0.236	0.868	0.790
P-value								
P (two-tailed)	<0.001	<0.001	<0.001	<0.001	<0.001	0.005	<0.001	<0.001
P-value summary	***	***	***	***	***	**	***	***
Significant? (alpha = 0.05)	Yes	Yes	Yes	Yes	Yes	Yes	Yes	Yes
Number of XY pairs	32	32	32	32	32	32	32	32

Consequently, we analyzed the peak times for patient visits between morning (8:00 AM-15:00 PM), afternoon (15:00 AM to 22:00 PM), and night (22:00 PM to 8:00 AM) shifts. As expected, the number of visits was substantially lower as the day progressed, with a median of 1621 patients in the morning hours, 800 patients in the afternoon, and 199 patients overnight across all months. The difference between morning patient influx in comparison to afternoon and night patient influx was found to be statistically significant (p < 0.001 for both comparisons). Interestingly, patient influxes were positively correlated in a proportional trend per month (Table 2).

**Figure 1:**
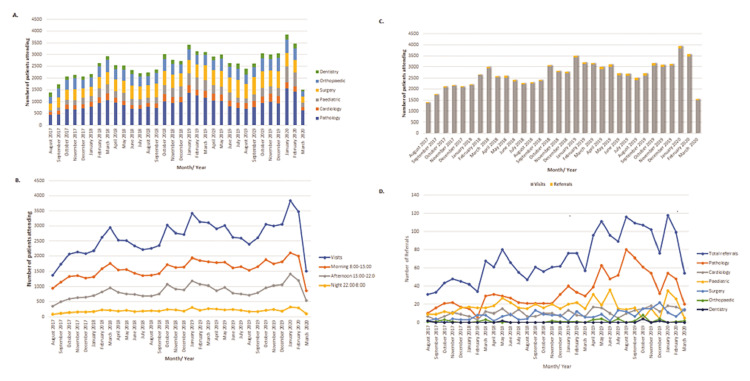
Specialty demand fraction against the total number of patients who visited across the months of analysis (A), total visit trend for the time period of the analysis and the time shifts (B), referrals/visits per month across the time span of the data collection (C) (total referrals are represented as yellow boxes in contrast to the total number of patients who visited per month [gray boxes]), and total referral trend for the time period of the analysis and the breakdown per specialty (D)

Our analysis showed that only a median of 64 patients/month throughout the analyzed time period were referred to secondary or tertiary care. Thus, 97.3% (n = 81,317) of total visits were assessed and treated within the reference primary care center, with only 2.72% (n = 2275) of the total influx of patients being referred to a higher level of specialty care. Notably, the months with the highest patient influx were January 2020, February 2020, and January 2019. The months with the highest number of referrals were January 2020, August 2019, and May 2019 (Figure 1, Panels A-D).

Furthermore, we sought to understand the underlying dynamic between the referrals and the medical specialty to which these were referred to. The total number of referrals was significantly correlated with pathology, with an R-value of 0.94, and cardiology, with an R-value of 0.70. Other statistically significant correlations were identified between total referrals to surgery (R-value: 0.44) and pediatrics (R-value: 0.41). The p-values were <0.001 for pathology, <0.001 for cardiology, 0.011 for surgery, and 0.021 for pediatrics. Referral correlation was not significant with either orthopedic or dentistry specialties (Table 3).

**Table 3:** R-values (correlation analysis) were computed for each datapoint per specialty against the total number of referrals across all months. Two-tailed p-values were calculated for each Pearson correlation coefficient, with the confidence interval set at 95%. NS: Not significant.

	Total vs. Pathology	Total vs. Cardiology	Total vs. Pediatrics	Total vs. Surgery	Total vs. Orthopedics	Total vs. Dentistry
Pearson r						
r	0.941	0.697	0.406	0.442	0.294	0.125
95% confidence interval	0.815; 1.000	0.429; 0.964	0.065; 0.747	0.107; 0.776	-0.063; 0.650	-0.245; 0.495
R squared	0.885	0.486	0.165	0.195	0.086	0.016
P-value						
P (two-tailed)	<0.001	<0.001	0.021	0.011	0.103	0.495
P-value summary	***	***	*	*	NS	NS
Significant? (alpha = 0.05)	Yes	Yes	Yes	Yes	No	No
Number of XY pairs	32	32	32	32	32	32

The reasons for patient referral are summarized in Table 4 and included the severity or complexity of the patient situation, lack of a specific medical specialty in the primary care setting, and unavailability of the laboratory during the night (22:00 PM-08:00 AM).

**Table 4:** Reasons for referral to a secondary or tertiary (third-level) hospital from the emergency department of the primary health care center

Departments	Pathology (n = 1078)	Cardiology (n = 333)
Reasons for patient referral/total number	Fever of unknown origin (n = 484)	Angina (n = 141)
	Paresis (n = 128)	
	Gastrointestinal bleeding (n = 110)	Tachyarrhythmias (n = 91)
	Fluid and electrolyte disorders (n = 104)	Acute myocardial infarction (n = 55)
	Diabetic ketoacidosis (n = 89)	
	Pancreatitis (n = 49)	Pulmonary edema (n = 30)
	Cholecystitis (n = 42)	
	Seizures (n = 38)	Pericarditis (n = 16)
	Psychoses/Delirium (n = 34)	
Departments	General surgery (n = 278)	Orthopedics (n = 37)
Reasons for patient referral/total number	Traumatic brain injury (n = 52)	Specific fractures (open, hip, vertebral physeal, intra-articular, malleolar) (n = 21)
			Multiple injuries (fall, work/road traffic accident) (n = 38)	Complex hand injuries (n = 12)
	Undiagnosed abdominal pain (n = 36)			
	Acute appendicitis (n = 34)	Lower extremity deep venous thrombosis (n = 2)
	Chest trauma (n = 32)	
	Ophthalmological trauma (n = 28)	Spinal cord injury (n = 1)
	Peripheral vascular disorder (n = 27)	
	Burns (chemical/electrical) (n = 20)	Pathologic fractures (n = 1)
	Ileus (n = 11)	
	Departments	Pediatrics (n = 545)	Dentistry (n = 4)
Reasons for patient referral/total number	Fever of unknown origin (n = 238)	Broken or fractured teeth (n = 3)
	Gastroenteritis, food or drug poisoning (n = 173)	
	Respiratory distress (n = 56)	
	Undiagnosed abdominal pain (n = 41)	Abscesses (n = 1)
	Anaphylaxis (n = 37)	
	Bronchiolitis (n = 24)	
	Gastrointestinal foreign bodies (n = 8)	Soft tissue injuries (tongue/lips) (n = 2)
	Seizures (n = 5)	

Finally, we found that our referral diagnosis matched the final hospital diagnosis in 91% of cases (2071 out of 2275). The 204 missed cases were diagnosed as fever of unknown origin (n = 132), undiagnosed abdominal pain (n = 46), paresis (n = 14), seizures (n = 10), and pathologic fractures/spinal metastasis (n = 2).

## Discussion

A well-organized PHC system may demonstrate a critical role in cornering public health improvement having a huge impact on promotion, prevention, and collaboration with secondary and tertiary healthcare structures. We believe that this kind of primary health model may successfully reorientate its crucial importance among healthcare services. This study aims to record the causes that lead patients to visit the first 24-hour-a-day urban health center in Greece and how this structure can be appropriate to confront these medical cases and decongest the secondary and tertiary hospitals.

Although the role of primary care as a vital component and contributor to health systems has been well established in the literature, the impact of such a role on the Greek population by an urban PHC unit has not been well examined. To the best of our knowledge, a study focused on patient visits and management by the first urban PHC center in Greece with an emergency department working 24 hours a day has not been discussed in the related literature.

Our study revealed that pathological conditions were the most common reasons for ED visits, followed by general surgical and orthopedic conditions. The main causes were respiratory diseases, trauma such as lacerations, abrasions, and low back or cervical spine pain. In addition, there were more visits in the morning than in the afternoon or even overnight.

In a systematic review conducted by Hong et al. [4], the authors studied the establishment of various initiatives in developed countries worldwide in order to ameliorate public access to after-hours primary care and their impact on ED and primary care usage. The results of cross-sectional studies were not quite indicative. Individuals having a PHC physician who offered medical services during weekends and evenings were less likely to visit the ED by 1.9% over two years. Subjects with access to after-hours PHC were less likely to use the ED than those without such access, in both the pediatric and adult populations [4].

Villani and Mortensen [5] reported that subjects who used the emergencies for non-ED situations seemed to have almost the same likelihood of receiving a usual source of care that offered services on weekends or during evening hours as those who did not use the ED (35.1% vs. 38.5%). Another study conducted in the United States on pediatric clinics demonstrated that access to after-hours primary care was not associated with non-urgent ED utilization [6].

Particular specialties might need additional state support, considering the number of patient visits they absorbed during all the months assessed in our data collection process. We further pondered whether a particular shift received more visits per day because, in terms of cost-effectiveness and productivity, staff coverage might need to be increased to accommodate the increasing demand to ensure patient safety. For example, internal medicine and general surgery uniformly received a substantially greater influx of patients across all months, in contrast to others such as dentistry. This finding was further evident and strengthened by the presenting complaint analysis. During the follow-up of this study, it is of great importance to record and analyze the shift during which most referrals occur and the presenting complaints of these patients. These additional data will lead to more educated and evidence-based decisions regarding funding and personnel redistribution in the most cost-effective and productive manner.

An important result of our study was the fact that the majority of the patients (81,317 out of 83,592, 97.3% of the total visits) who attended the health center were managed in a timely manner, with only 2.72% of patients needing referral to another health center (secondary or tertiary).

Dy et al. [7] conducted a cross-sectional qualitative interview study of 32 patients who were transferred to tertiary care or their surrogate decision-makers. The authors found that patient safety, coordination of care, and patient-physician communication are important in patients’ judgments of hospital care quality. They also noted that many transfers might be avoided by improving the aspects of quality at the referring facility as subjects more often mentioned remediable dimensions of quality, such as communication, than dimensions intrinsic to the facility, such as lack of resources.

In contrast, Glass et al. [8] noted that a better approach to PHC for an employed, insured population through the provision of a workplace clinic does not necessarily guarantee a shift from more costly visits, such as emergency services or patient admissions, to primary care in the United States [8]. Another issue contributing to the problem is the lack of a good referral system consisting of organized and combined electronic databases between primary and secondary healthcare systems [9-11].

In addition, all referrals to other centers (secondary or tertiary) were due to severe or complex cases, lack of specific surgical or other medical specialties, or unavailability of the laboratory during the night.

The main aim of every healthcare system, within the boundaries of the social state, is to secure the health of its population and the improvement of the level of prosperity and quality of life. The Greek healthcare system, with the aforementioned legislative regulations, sought to upgrade its PHC services [9].

The small percentage (2.7%) of our referrals was a particularly optimistic finding as it demonstrates that healthcare centers, as a public structure of healthcare service provision, can substantially decongest the secondary healthcare system, serving the purpose of PHC, which is not only the prevention and promotion of health but also the provision of healthcare services, through the effective management of emergent incidents and the promotion of PHC. We believe that the studied unit has the following advantages: a 24/7 schedule, good organization, easy accessibility (nearby public transportation), competence of personnel, and short waiting times.

Moreover, as this study represents the first analysis of the PHC sector in Greece, it provides preliminary evidence in support and promotion of the vast benefits of a robust primary care sector. Alleviation of unnecessary referrals to an already “fatigued” tertiary care system, sensible utilization of funding resources, and a reduction in patients’ waiting times focusing on patient safety and optimum health care are the most highlighted benefits of an empowered nationwide PHC sector. Concerning the time of waiting issue, the current literature seems to confirm the existence of a negative correlation between the waiting time, mainly registration and consultation, and the patient's feeling of satisfaction. Studies from different countries showed that the ED waiting time could be ranged from 20 to 74 minutes [12,13]. However, the above studies indicate that waiting time varies due to multiple patient populations, low response rates of questionnaires, and different physicians’ specialties.

Nevertheless, the topic of patient satisfaction versus waiting time and also the attitude of healthcare providers, are not the main objectives of our study.

However, the PHC system has not been fully developed, and patients still have a lot of difficulties with access, continuity of care, and coordination, as well as service comprehensiveness. Beyond a public system, private providers also provide ambulatory care [9,14]. Currently, there is no gatekeeping mechanism managing the referral system, but the new Primary Care Plan announced in 2017 aims to establish first-contact, decentralized local primary care units staffed by multidisciplinary teams, which will also take on a gatekeeping role [9].

Recently, Kringos et al. [15], suggested that the average length of consultation could be a useful tool for monitoring PHC outcomes. The authors showed that a high level of quality regarding PHC evidence and information in most of the European countries on the field of contribution was insufficient. They also stressed that although the primary health organization theoretically has good potential, the effectiveness of the system may need to be targeted for improvement actions in policy and financial characteristics. In addition, Macinko et al. [16] found that a well-established health system seems to have a considerable influence on health consequences globally. The authors also noted that numerous European countries have re-educated district physicians and primary care specialists doctors into general practitioners across Europe and introduced gatekeeping [17].

However, as our study has numerous limitations, our findings should be interpreted cautiously. First, as with all observational studies, there remains a risk of residual confounding, although our models were adjusted using available data on practice and population characteristics. Second, after the period of March 2020, our healthcare medical center has been determined as COVID-19/SARS-CoV-2 referral point. Consequently, all the following visits were only related to specific pathological conditions. Third, our main purpose is to record the kind of medical cases presented at this PHC and the efficiency of appropriate and immediate responses toward these cases in order to avoid any meaningless referral to hospitals, where serious cases are referred, providing hospitals with the opportunity to focus on their pivotal role. It is clear that these findings could not be generalized and further investigation is needed. We firmly believe that through this study, we could set the frame in order for more relative studies to be carried out with a larger number of patients and data among other PHCs of Greece and other countries worldwide. It seems that if the PHC system is well organized, this could be extremely beneficial for a country’s hospitals.

Despite several nationwide attempts to improve and homogenize the PHC system, Greece still lacks a viable, policy-based model of consolidated services. Sifaki-Pistolla et al. [18] stated that despite the reforms and improvements, levels of incorporation remain low mainly in PHC services, and they pointed out that issues in health promotion and preventive services continue to be unsolved. They also proposed that both PHC units and the Ministry of Health need to keep a systematic monitor integration with respect to patient quality healthcare improvement.

While groundbreaking and pioneering, as it is the first study to address the importance of a structured PHC system in Greece, the present study is restricted to a degree by the obvious limitation of collecting and analyzing data from a single PHC center, albeit centrally located. The practical implications of the present results would exponentially increase if more PHC centers recorded and reported their data in a central database. In that scenario, a bird’s eye view would be obtained to critically assess the benefits and drawbacks of having a stronger PHC medical base and consequently enable the Ministry of Health to redirect state funding in the most cost-effective and appropriate manner to ensure optimum health care and safety for our patients.

There is a considerable indication that improved or superior access to PHC is related to decreased utilization of the ED, reduced patient admissions, surgical operations, and fewer costs [19-21]. However, according to other studies [22-24], readily accessible PHC, under certain terms, would appropriately shift health care away from more intensive and expensive provided services in EDs, specific departments, and hospitality.

Recently, Sanderson et al. [25] suggested a healthcare team-based approach model centralized around the individual patient. They mentioned that a multicare approach recognizes that each patient possesses differing medical needs, moving care from generalized medical treatments to individualistic care. Under these circumstances, a patient-centered medical home model can result in enhanced patient outcomes, satisfaction, quality, reduction in costs, and improved work life of providers and staff.

To date, several theoretical models have been developed for the organization of primary care [26-30]. Based on the assessment of PHC in Greece, a number of recommendations have been formulated into an action plan to improve PHC, such as the following:

(1) A unified acceptable definition of PHC, as well as a health and general attitude and approach to all levels of medical services, was developed. These definitions should be adapted to Greek conditions, taking into account the health needs of the Greek population, the value system of Greek society, and, of course, patient expectations. (2) There is a need for coordinated and patient-centered health care that provides accessible team-based, prevention-focused primary care and a holistic patient medical approach. (3) The interface of the PHC system should be organized within the health system as a whole, with particular reference to its bridging with public health services, hospital services, social care services, and governmental organizations. (4) PHC physicians should develop disease-specific protocols and clinical guidelines. Continuity of care should be facilitated by medical records. (5) Continuing education of PHC professionals and support for the clinical practice and basic skills of the health providers should be provided in healthcare structures and units. An assessment of training needs for PHC professionals is recommended, and short training courses should be developed and implemented to obtain the core competencies stated in the job description. Policies for the education and training of health professionals should address the imbalance between generalists and specialists and serve to increase the flow of general practice training. (6) Chronic disease prevention, potential risk factors monitoring, and identification of people at risk for developing chronic conditions should be performed. (7) Providing PHC providers with education and tools to prioritize prevention during patient encounters is necessary. (8) Assessing the performance and quality of health professionals and services with the involvement of both patients and individuals employed in structures and health units is needed. (9) A system of compensation for health care professionals that includes incentives in order to improve performance should be established.

## Conclusions

The objective of PHC is not only the prevention and promotion of public health but also the provision of healthcare services among the general population of different economic statuses, through the effective management of emergency incidents and the promotion of patients’ care in this field. It is of great importance to focus on strengthening PHC. The motivated and qualified staff of PHC institutions constitute the cornerstone of this system. Improving patient satisfaction, managing patient expectations, and effectively solving problems are essential factors. Improvement in the management of PHC institutions increases competitiveness; therefore, setting the priorities of value creation and selection of competitive abilities are essential both for the patient and the healthcare system. The development of urban PHC centers with 24-hour-a-day services could be crucial for the clinical effectiveness and decongestion of the secondary and tertiary healthcare systems.
